# A novel microfluidic model can mimic organ-specific metastasis of circulating tumor cells

**DOI:** 10.18632/oncotarget.9382

**Published:** 2016-05-15

**Authors:** Jing Kong, Yong Luo, Dong Jin, Fan An, Wenyuan Zhang, Lilu Liu, Jiao Li, Shimeng Fang, Xiaojie Li, Xuesong Yang, Bingcheng Lin, Tingjiao Liu

**Affiliations:** ^1^ College of Stomatology, Dalian Medical University, Dalian, China; ^2^ Faculty of Chemical, Environmental and Biological Science and Technology, Dalian Technology University, Dalian, China; ^3^ Department of Biochemistry and Molecular Biology, Liaoning Provincial Core Lab of Glycobiology and Glycoengineering, Dalian Medical University, Dalian, China; ^4^ Department of Biotechnology, Dalian Institute of Chemical Physics, Chinese Academy of Sciences, Dalian, China

**Keywords:** microfluidic, metastasis, circulating tumor cells, multi-organ, bionic model

## Abstract

A biomimetic microsystem might compensate costly and time-consuming animal metastatic models. Herein we developed a biomimetic microfluidic model to study cancer metastasis. Primary cells isolated from different organs were cultured on the microlfuidic model to represent individual organs. Breast and salivary gland cancer cells were driven to flow over primary cell culture chambers, mimicking dynamic adhesion of circulating tumor cells (CTCs) to endothelium *in vivo*. These flowing artificial CTCs showed different metastatic potentials to lung on the microfluidic model. The traditional nude mouse model of lung metastasis was performed to investigate the physiological similarity of the microfluidic model to animal models. It was found that the metastatic potential of different cancer cells assessed by the microfluidic model was in agreement with that assessed by the nude mouse model. Furthermore, it was demonstrated that the metastatic inhibitor AMD3100 inhibited lung metastasis effectively in both the microfluidic model and the nude mouse model. Then the microfluidic model was used to mimick liver and bone metastasis of CTCs and confirm the potential for research of multiple-organ metastasis. Thus, the metastasis of CTCs to different organs was reconstituted on the microfluidic model. It may expand the capabilities of traditional cell culture models, providing a low-cost, time-saving, and rapid alternative to animal models.

## INTRODUCTION

Metastasis represents a highly organized and organ-specific process, with multiple destinations such as the lung, liver, and bone [1–4]. There are multiple steps of hematogenous metastasis, including degradation of the extracellular matrix, intravasation into blood vessels, surviving in the circulation, and extravastion into distant organs. Individual cancer cells involved in this metastasis that detach from the original tumor and circulate through the blood stream are called circulating tumor cells (CTCs), and are considered responsible for the metastasis [5].

Experimental metastatic models not only improve our understanding of the mechanisms of metastasis, but also serve as a platform to screen anti-metastatic drugs. Animal models has been considered the standard method to study metastasis [6–8]. Generally, there are two ways to model organ specific metastasis of human cancers using laboratory mice. The first is orthotopic transplantation of human cancer cells into mice. Using this methodology, it may take several months to form histologically detectable metastasis [9–12]. Alternatively, cancer cells can be injected directly into the systemic circulation of animals to mimic CTCs and accelerate metastasis. Metastasis usually occurs after several weeks. Overall, CTC metastasis to multiple organs can be well reproduced by animal models. However, these models involve complicated procedures, and are expensive and time-consuming, resulting in the high cost of pharmaceuticals and delayed development of new drugs [7, 13–18]. In contrast, most *in vitro* metastatic models involve simple procedures, and are inexpensive and time-saving, such as the widely-used Transwells^®^. However, by culturing cells in a static condition, these Transwells^®^ are unable to accurately reproduce the dynamic adhesion of CTCs to endothelium [19, 20].

A biomimetic microsystem reproducing the dynamic CTC metastasis to multiple organs may offer a solution to overcome the disadvantages of animal models, while facilitating the simple, inexpensive, and time-saving advantages of the Transwell^®^ system. As a new technology, microfluidics creates new opportunities for spatial and temporal control of cell growth and stimuli. Microfabricated devices have been developed to facilitate both applied and basic research concerning the biology of cells, tissues, even organs [21–26]. It has been emerging as an ideal platform to construct biomimetic models. These integrated microsystems can replicate the complex physiological functions of living organs, such as the functional alveolar capillary interface of the human lung. Some mcrofluidic-based platforms have been developed to replicate cancer invasion and metastatic processes [27–32]. However, currently there have been no reports that have described engineering of integrated microsystems that replicate the complex CTCs metastasis by incorporating multiple organs, microvessels, and CTCs, and then placing them in a dynamic microenvironment.

In the present study, we describe the development of a microfluidic model to mimic multiple organ metastasis of CTCs. Using this microfluidic model, we modeled the potential of breast and salivary gland cancer cells to metastasize to the lung. When compared with traditional nude mouse model, similar results to those obtained from the microfluidic model were revealed. It demonstrated that this biomimetic model facilitated the rapid study of lung metastasis of CTCs. When we used this biomimetic model to evaluate the ability of antimetastatic agents to inhibit lung metastasis of breast cancer cells, similar effects were observed for both the microfluidic model and nude mouse model. Furthermore, the microfluidic model was used to mimick liver and bone metastasis of CTCs and confirmed the potential for research of multiple-organ metastasis.

## RESULTS

### Reconstitution of the microfluidic multiple organ model

**Figure 1:**
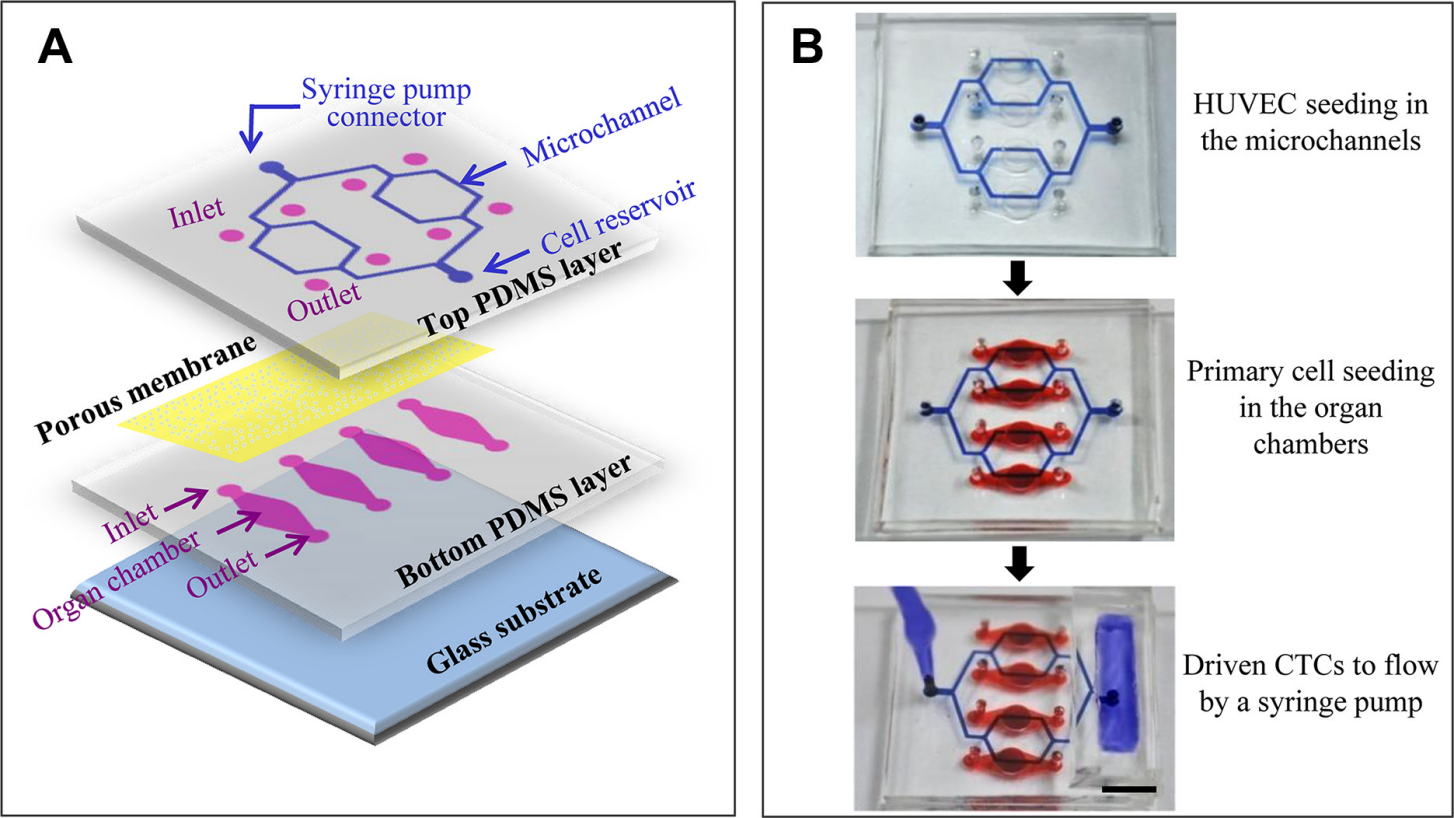
Reconstitution of the microfluidic model (**A**) The design of the microfluidic model. This model is composed of four layers: one layer of glass substrate, two layers of a PDMS membrane, and one porous membrane. The porous membrane is sandwiched between two PDMS layers. There are four parallel branched microchannels mimicking vascular microvessels on the top PDMS layer. These microchannels join together at the syringe pump connecter at one end and the cell reservoir at the other end. There are four organ chambers on the bottom PDMS layer. After the top and bottom PDMS layers were sealed together, the inlet and outlet of each organ chamber were punched through from the top to the bottom layer. (**B**) Photos of the established microfluidic model. Scale bar = 5 mm.

The microfluidic model was composed of four layers: one layer of glass substrate, two layers of a PDMS (polydimethylsiloxane) membrane, and one layer of a porous polycarbonate membrane with 3 μm pore size and 6 μm thickness (Figure 1A). PDMS is widely used in microfluidic platforms for biological research because of its good biocompatibility and gas permeability. The bottom PDMS layer contained four separate organ chambers. Each chamber had its own inlet and outlet. The top PDMS layer contained four parallel microchannels (60 μm height and 400 μm width) that joined together at the cell reservoir at one end and at the syringe pump connector at the other end. The four microchannels were located on the top of four organ chambers, respectively. A transparent polycarbonate membrane separated each chamber and microchannel. The established microfluidic model was shown in Figure 1B. A syringe pump was connected to the microchannels to drive the flow of CTCs via the syringe pump connector.

**Figure 2:**
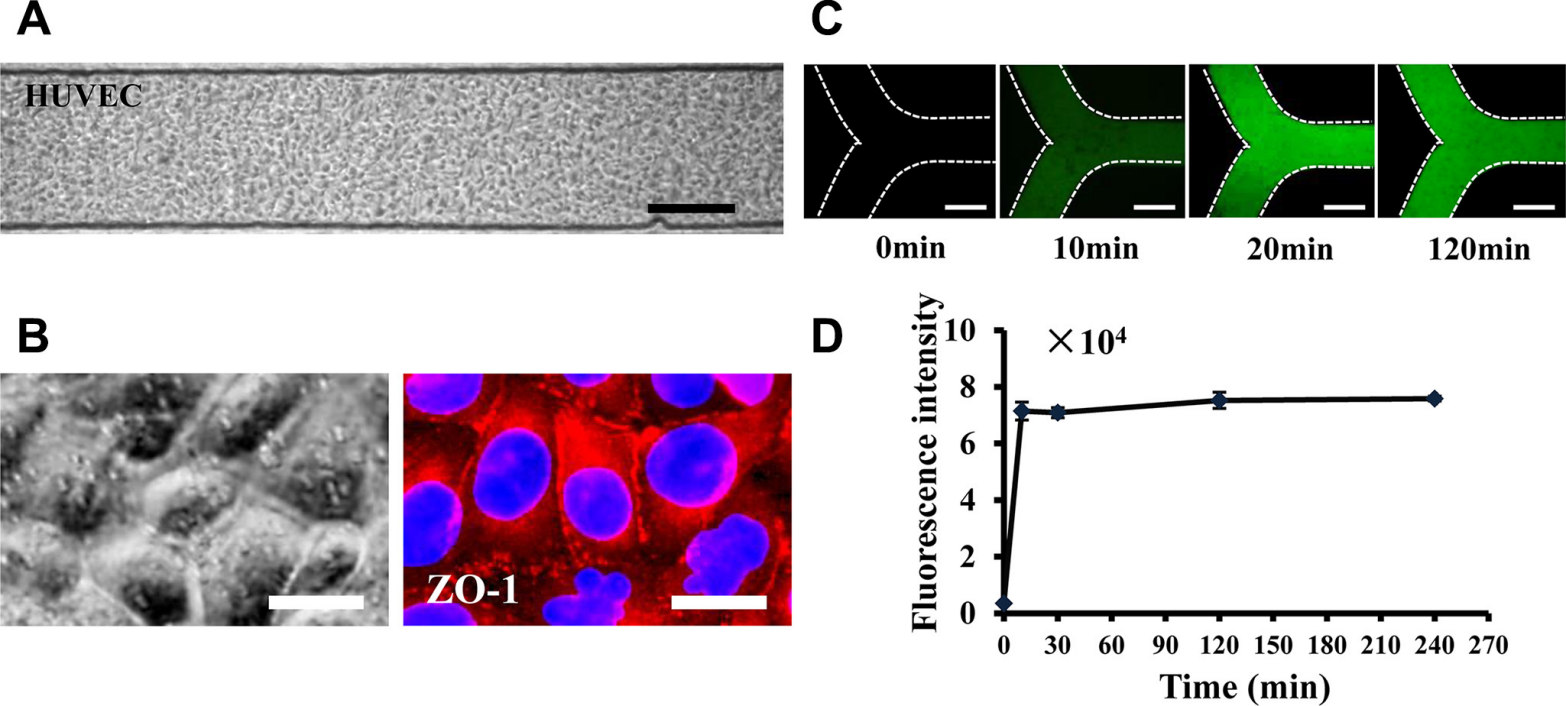
Mimicking the vascular endothelial barrier on the microfluidic (**A**) HUVEC cells form a monolayer barrier in the microchannels. (**B**) (Left) Bright field of the HUVEC layer at a high magnification. (Right) ZO-1 immunostaining indicates the tight junctions between HUVEC cells. Scale bar = 25 μm. (**C**) FITC-dextran (10 kDa) was loaded into the organ chambers. Images of FITC-dextran diffusing through the HUVEC layer into the microchannels were captured at different times. Scale bar = 250 μm. (**D**) Quantitation of fluorescence intensity to assess the permeability of the HUVEC layer (*n* = 3).

In order to mimic the endothelial barrier of blood vessels, human umbilical vein endothelial cells (HUVEC) were seeded in the microchannels, pre-coated with Cultrex Basement Membrane Extracts (BME), a substitution of extracellular matrix, via the cell reservoir at first. Primary cells were loaded into the organ chambers via individual inlets. HUVEC cells attached to the porous membrane and formed a monolayer barrier, to mimic the barrier *in vivo* (Figure 2A). ZO-1 expression was confirmed, indicating the tight junction of HUVEC cells (Figure 2B). To characterize the permeability of the HUVEC barrier, fluorescein isothiocyanate-dextran (10 kDa) was introduced into each organ chamber. The fluorescein diffused from the chamber into the microchannels with time (Figure 2C). Quantitative analysis demonstrated that the fluorescence intensity increased with time, reached a steady state after 20 minutes, and remained stable during the time of the experiment (Figure 2D).

### Chemokine-induced metastasis in the microfluidic model

**Figure 3:**
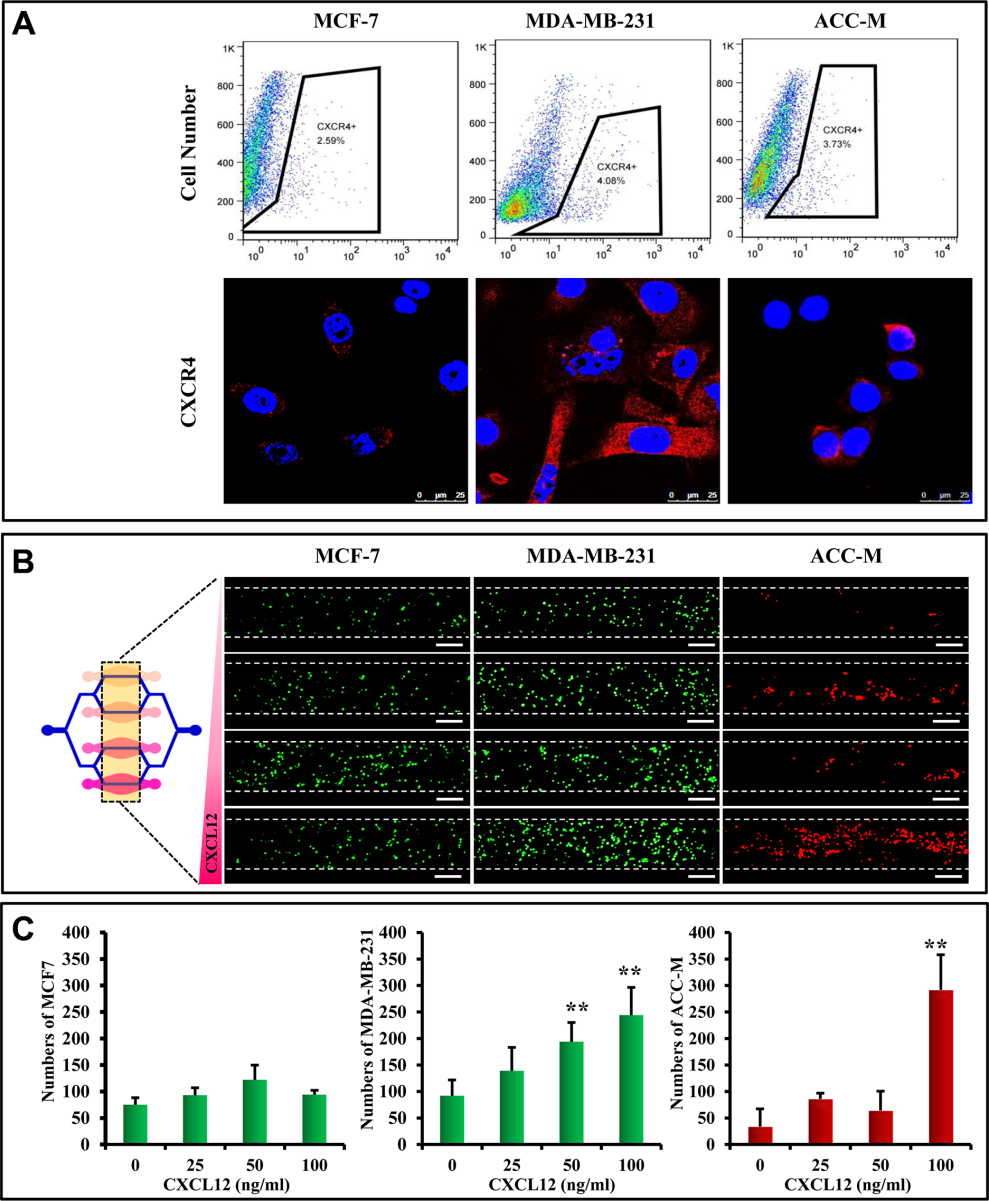
Chemokine-induced metastasis of CTCs in the microfluidic model (**A**) CXCR4 expression on MCF-7, MDA-MB-231, and ACC-M cells detected by flow cytometry and immunofluorescent staining. CXCR4 (red) is expressed on the cell membrane, and DAPI (blue) indicates cell nuclei. Scale bar = 25 μm. (**B**) CXCL12-induced CTC metastasis in the microfluidic model. The concentrations of CXCL12 were 0 ng/mL, 25 ng/mL, 50 ng/mL, and 100 ng/mL from the upper to the lower chambers. MCF-7 cells (left) and MDA-MB-231 cells (middle) are labeled with CellTracker (green) (Life Technologies Corp., Gaithersburg, MD, USA), and ACC-M cells (right) are labeled with CellTracker (red) (Life Technologies Corp., Gaithersburg, MD, USA). Scale bar = 200 μm. (**C**) Quantification of adhesive cell numbers. The number of MDA-MB-231 cells increased significantly with 50 ng/mL and 100 ng/mL of CXCL12 (**p < 0.01, *n* = 8 per group). However, adhesion of ACC-M cells significantly increased only with 100 ng/mL CXCL12 (**p < 0.01, *n* = 8 per group).

Chemokine CXCL12 is involved in metastasis via bonding to CXCR4, which is expressed on cell membranes in a wide variety of tumors [33, 34]. Furthermore, flow cytometry and immunofluorescent staining with anti-CXCR4 antibody indicated that MCF7, MDA-MB-231, and ACC-M cells expressed CXCR4 (Figure 3A). We therefore determined CXCL12-induced metastasis of MCF7, MDA-MB-231, and ACC-M cells using the microfluidic model. It was found that some of the flowing tumor cells stopped and attached to HUVEC cells when they passed over the top of the organ chambers containing CXCL12. The number of attached tumor cells gradually increased with CXCL12 concentration (Figure 3B and 3C). Furthermore, the arrested MDA-MB-231 cells significantly increased with 50 ng/mL and 100 ng/mL of CXCL12, while the arrested ACC-M cells significantly increased only with 100 ng/mL CXCL12. In contrast, the arrested MCF7 cells increased with a high concentration of CXCL12, but not in a significant manner.

### Lung metastasis in microfluidic model

**Figure 4:**
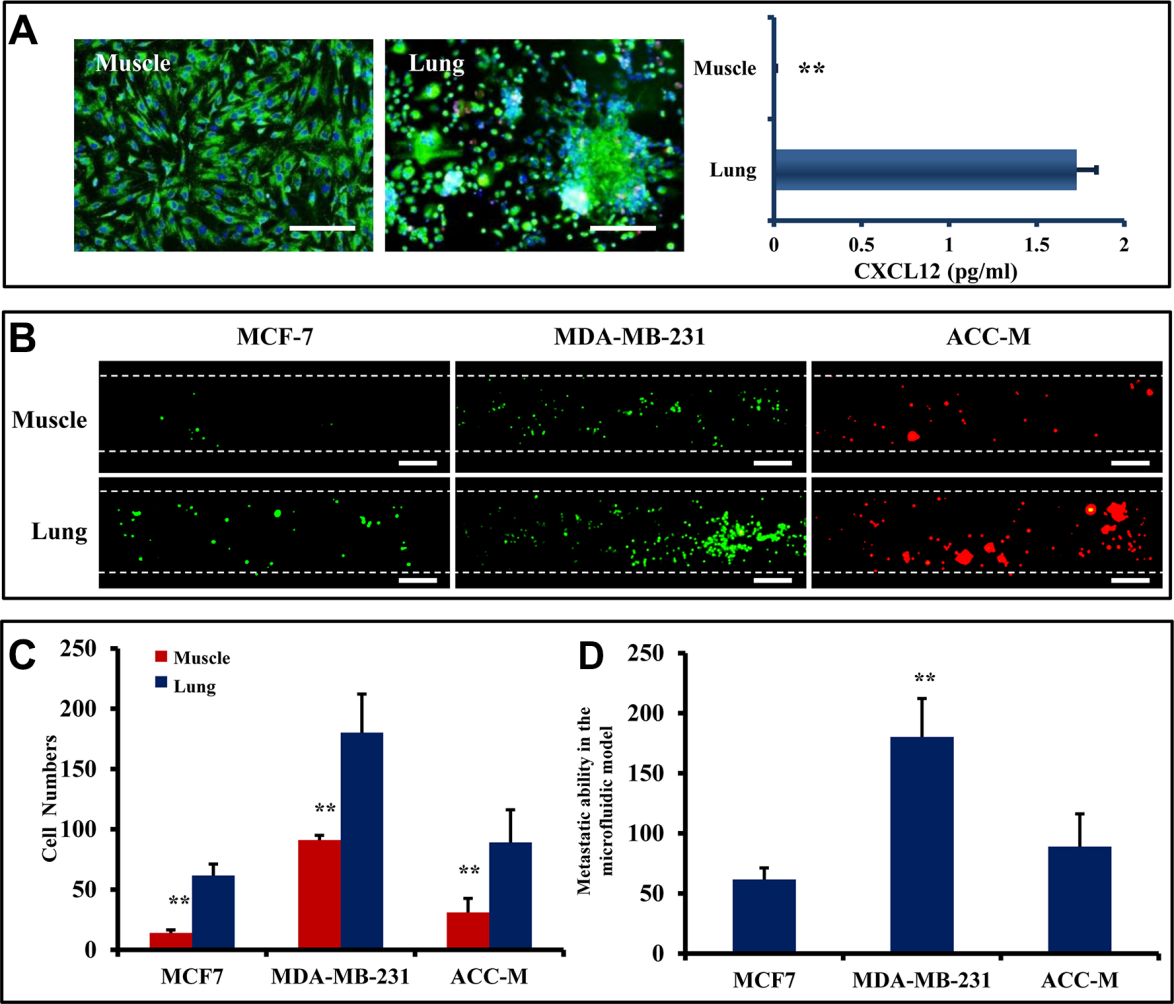
Lung metastasis in microfluidic model (**A**) (Left) Primary cells of lung and muscle were isolated from SD-rat and stained with Rh-123 (green) and Hoechst 33 342 (blue) showed 95% viability. Scale bar = 250 μm. (Right) Elisa assay for the detection the CXCL12. Lung- derived CXCL12 is more than muscle-derived CXCL12 (**p < 0.01, *n* = 3). (**B**) Image of adhesion cells. The cell lines are MCF7 (Green), MDA-MB-231 (Green) and ACC-M (Red) from left to right. Scale bar = 200 μm. (**C**) Statistical analysis showed that the numbers of arrested MCF7, MDA-MB-231, and ACC-M cells by lung were significantly higher than those arrested by muscle cells (**p < 0.01, *n* = 8). (**D**) Lung metastatic capability of MCF-7, MDA-MB-231, and ACC-M cells assessed using the microfluidic model. MDA-MB-231 showed the highest metastatic capability among the three cell lines (**p < 0.01, *n* = 8 per group).

Lung metastatic model is widely used in the study of both breast and salivary gland cancers [35, 36]. The capabilities of lung metastasis of MCF7, MAD-MB-231, and ACC-M cells were assessed in the microfluidic model. Mixed primary cells isolated from the lung of SD rat were used to represent the lung. Primary muscle cells were used as a control. These primary cells showed good viability (Figure 4A). Quantitative analysis of CXCL12 expression by ELISA showed that CXCL12 was expressed in primary lung cells, but showed markedly low expression in primary muscle cells (Figure 4A). The primary cells were seeded into the organ chambers of the microfluidic model, respectively. When MCF7, MDA-MB-231, and ACC-M cells flowed over the cell organ chambers, different numbers of tumor cells stopped and attached to HUVEC (Figure 4B). Statistical analysis showed that the numbers of arrested MCF7, MDA-MB-231, and ACC-M cells by lung cells were significantly higher than those arrested by muscle cells (Figure 4C). In addition, MDA-MB-231 cells showed the strongest lung metastatic capability among the three cancer cell lines tested in the microfluidic model (Figure 4D).

### Physiological similarity of the biomimetic microfluidic model to animal models

**Figure 5:**
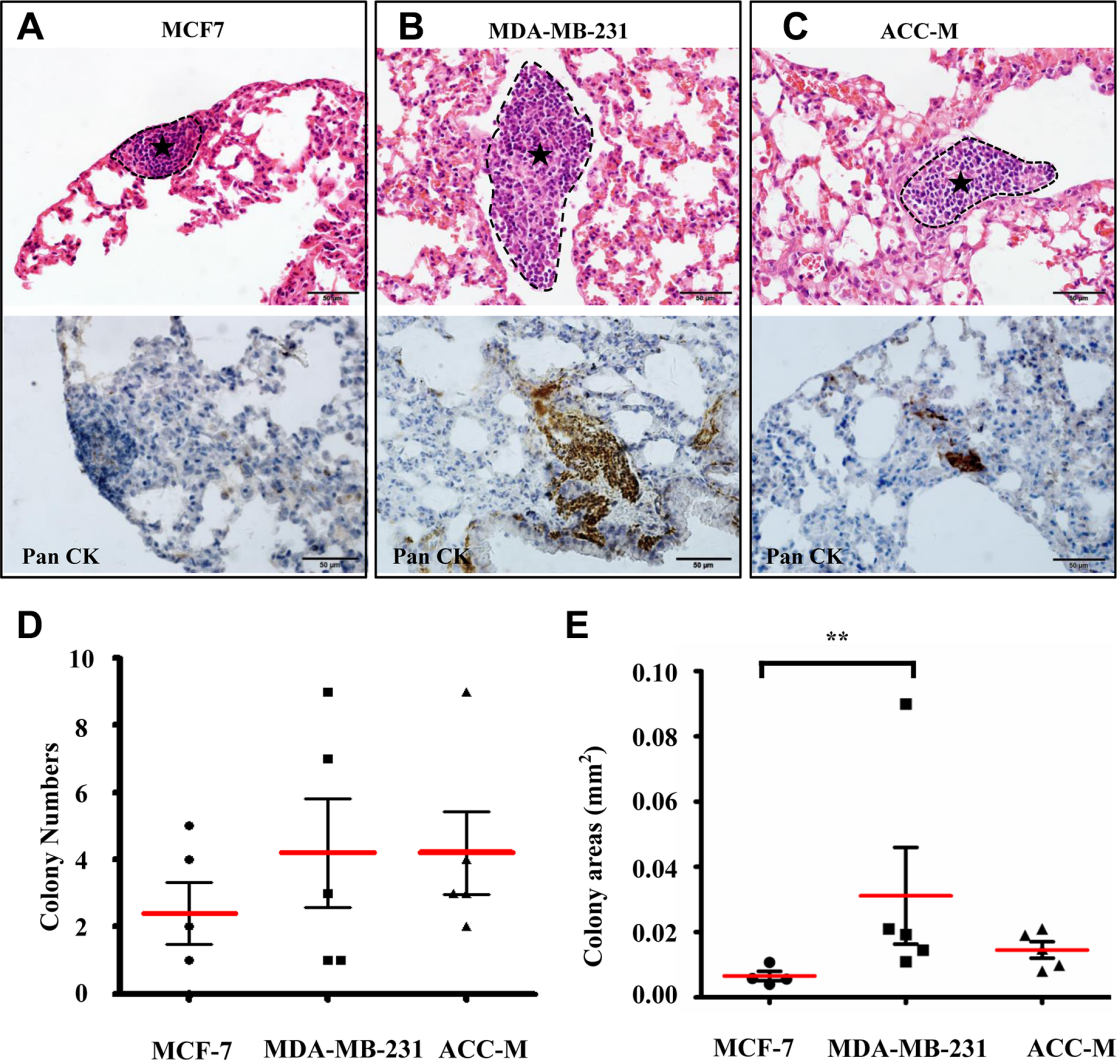
Physiological similarity of the microfluidic model to the animal model (**A**–**C**) Typical metastatic colonies of MCF-7, MDA-MB-231, and ACC-M cells after HE staining and anti-human Pan CK immunostaining in the lung of nude mice. The metastatic colonies are circumscribed by black dotted lines and indicated by asterisk. Scale bar = 50 μm. (**D**) The metastasis rates and the colony numbers of MCF7, MDA-MB-231, and ACC-M, respectively. (**E**) The metastasic colony areas of MCF-7, MDA-MB-231, and ACC-M cells. The colony areas of MDA-MB-231 cells were significantly higher than those of MCF-7 cells (** *p* < 0.01, *n* = 5 per group).

To further investigate the physiological similarity of the microfluidic model to animal models, we performed lung metastatic assays using the traditional nude mouse model and compared the metastatic capabilities of MCF7, MDA-MB-231, and ACC-M cells using the microfluidic and animal models. Fifteen nude mice were equally divided into three groups, and MCF7, MDA-MB-231, and ACC-M cells were inoculated into the tail vein. After one month, lung metastasis of each mouse was histologically examined. As shown in Figure 5A–5C, metastatic colonies of various sizes formed in lung. Consistent with MCF7 cells showing the lowest metastatic capability using the microfluidic model, the metastasis rates of MCF7, MDA-MB-231, and ACC-M were 80%, 100% and 100%, respectively. The colony numbers of MDA-MB-231 and ACC-M were also higher than that of MCF7, but not significantly (Figure 5D). We compared the colony areas per lung of MCF7, MDA-MB-231, and ACC-M cells. It was found that MDA-MB-231 showed the highest metastatic capability among the three cancer cell lines (*p* < 0.05) (Figure 5E), which was consistent with that assessed by the microfluidic model (Figure 4D).

### Inhibition of lung metastasis in the microfluidic model and mouse model

**Figure 6:**
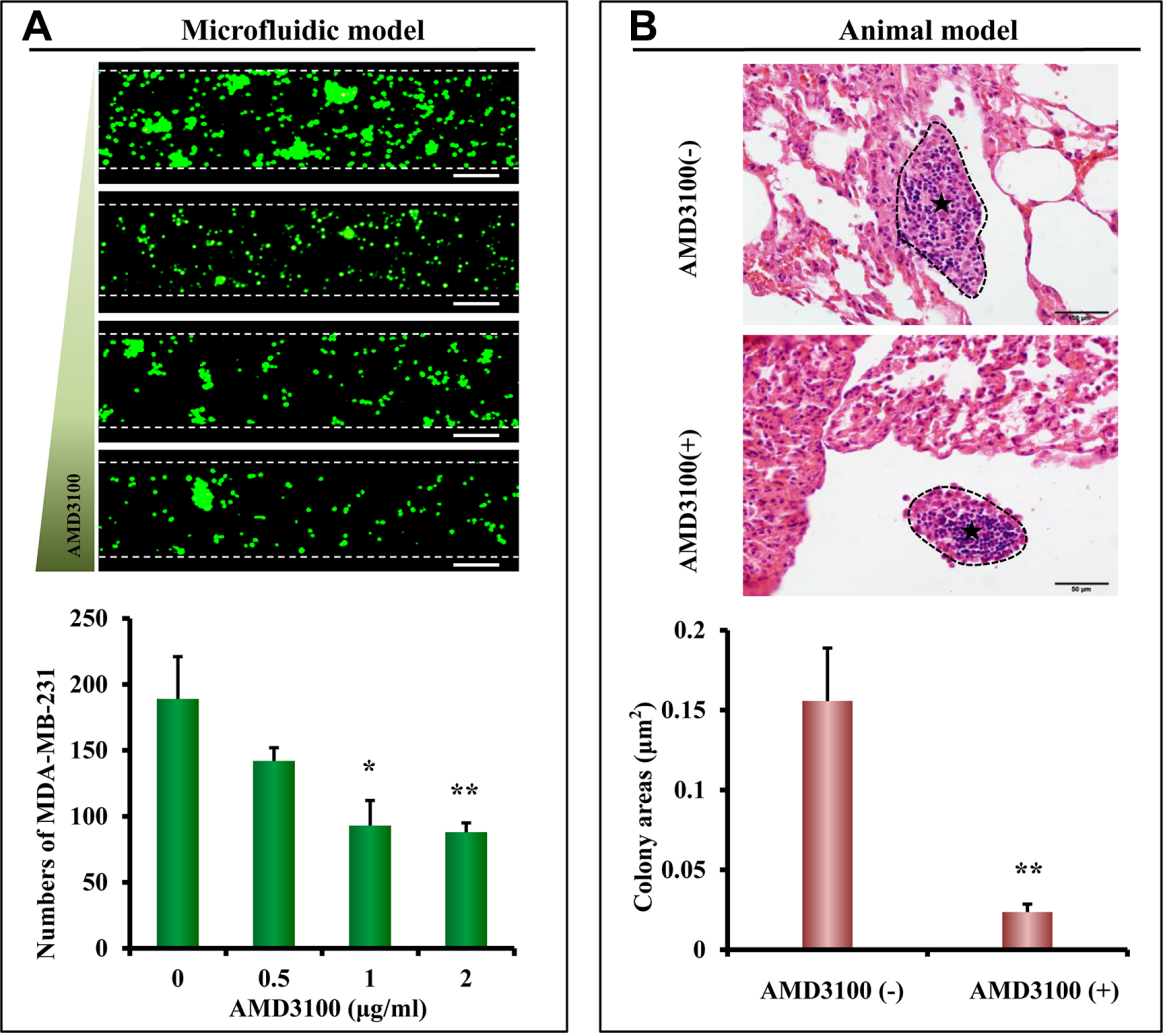
Inhibition of lung metastasis in the microfluidic model and nude mouse model (**A**) Different concentrations of AMD3100 were used to inhibit the metastasis of MDA-MB-231 cells in the microfluidic device. Scale bar = 200 μm. Using the microfluidic model, quantitative analysis showed that 1 μg/mL and 2 μg/mL of AMD3100 significantly inhibited lung metastasis of MDA-MB-231 cells (*p < 0.05, **p < 0.01, *n* = 8 per group). (**B**) As assessed by colony areas, AMD3100 significantly inhibited lung metastasis of MDA-MB-231 cells in nude mice (**p < 0.05, *n* = 5 per group). The metastatic colonies are circumscribed by black dotted lines and indicated by asterisk.

The microfluidic model was then used to evaluate the inhibitory effect of anti-metastatic drugs. AMD3100, a small molecule antagonist of CXCR4, has been reported to inhibit CXCL12-induced tumor metastasis [37, 38]. When AMD3100 was tested using the microfluidic model with primary lung cells in organ chambers, the number of cancer cells attached to the HUVEC layer was reduced gradually by increasing the AMD3100 concentration (Figure 6A). These results suggest that AMD3100 significantly inhibited lung metastasis of MDA-MB-231 cells (*p* < 0.05). Nude mice were then treated with AMD3100, and the results of this *in vivo* model compared with the results obtained from the microfluidic model. In a similar manner as the microfluidic model, the colony areas significantly decreased with AMD3100 treatment (*p* < 0.05) (Figure 6B). This suggests that AMD3100 inhibited lung metastasis of MDA-MB-231 cells in nude mice, as assessed both by metastatic areas and colonies per lung section.

### Liver and bone metastasis in the microfluidic model

**Figure 7:**
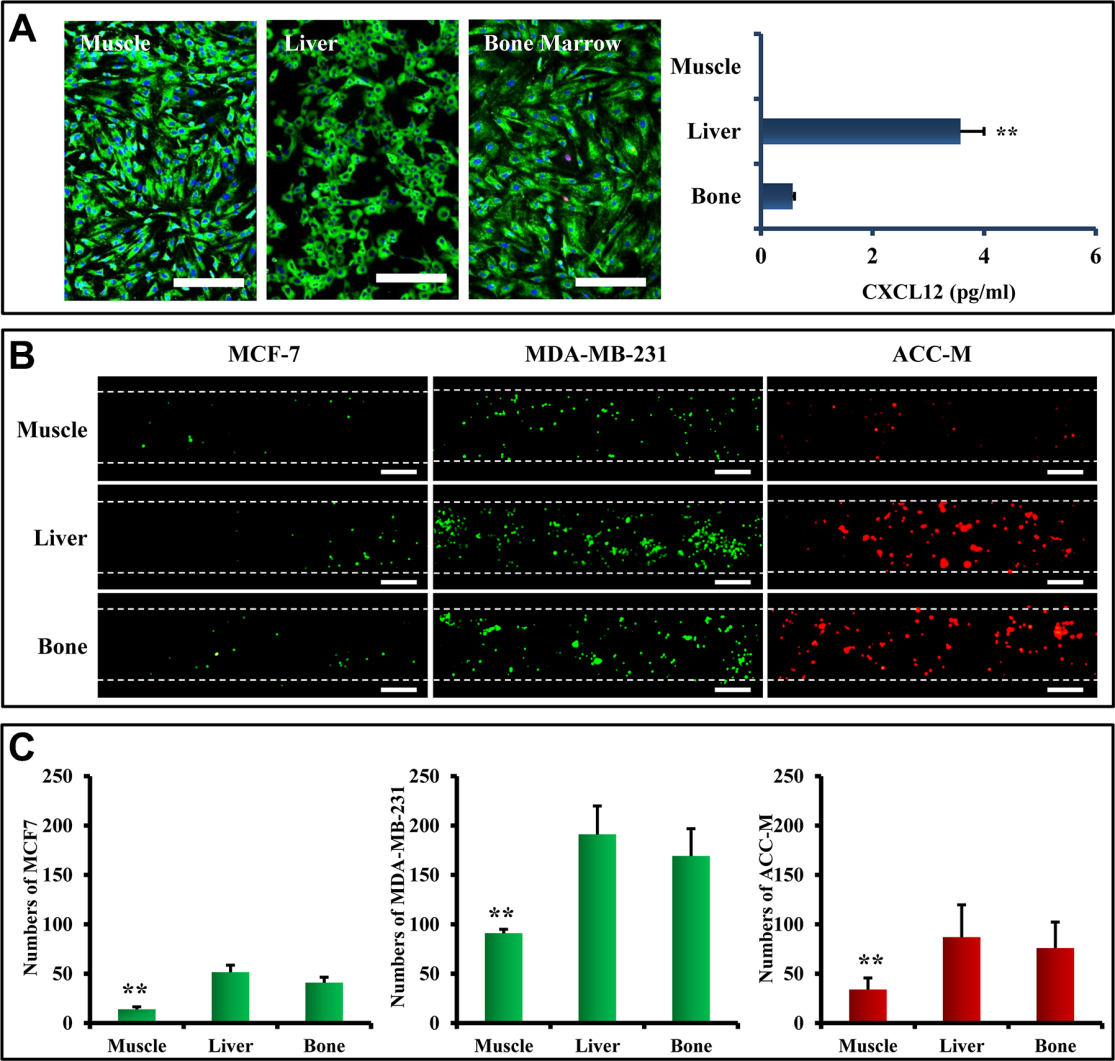
Liver and bone metastasis of CTCs in the microfluidic model (**A**) (Left) Primary cells of muscle, liver, and bone marrow were isolated and stained with Rh-123 (green) and Hoechst 33 342 (blue). Most of the cells showed good viability. Scale bar = 250 μm. (Right) Determination of CXCL12 secretion of primary cells by ELISA. Except for muscle cells, primary cells from liver and bone secreted CXCL12 into their culture medium. (**B**) Flowing MCF-7, MDA-MB-231, and ACC-M cells were arrested by primary cells. Scale bar = 200 μm. (**C**) The arrested tumor cells showed an organ-specific pattern. Statistical analysis showed that the numbers of arrested MCF7, MDA-MB-231, and ACC-M cells by liver, and bone marrow were significantly higher than those arrested by muscle cells (**p < 0.01, *n* = 8).

In addition to the lung, liver and bone marrow are common metastatic sites in both breast and salivary gland cancers [35, 36]. To demonstrate the potential of assessing multi-organ metastasis in the microfluidic model, primary cells isolated from liver and bone marrow were used to represent specific organs. Primary muscle cells were used as a control. These primary cells showed good viability of over 90% (Figure 7A). Quantitative analysis of CXCL12 expression by ELISA showed that CXCL12 was expressed in primary liver and bone marrow cells, but showed markedly low expression in primary muscle cells (Figure 7A). The three types of primary cells were seeded into the organ chambers of the microfluidic model, respectively, so that each chamber represented a specific organ. When MCF7, MDA-MB-231, and ACC-M cells flowed over the cell organ chambers, different numbers of tumor cells stopped and attached to HUVEC (Figure 7B). Statistical analysis showed that the numbers of arrested MCF7, MDA-MB-231, and ACC-M cells by liver, and bone marrow cells were significantly higher than those arrested by muscle cells (*p* < 0.01) (Figure 7C).

## DISCUSSION

**Figure 8:**
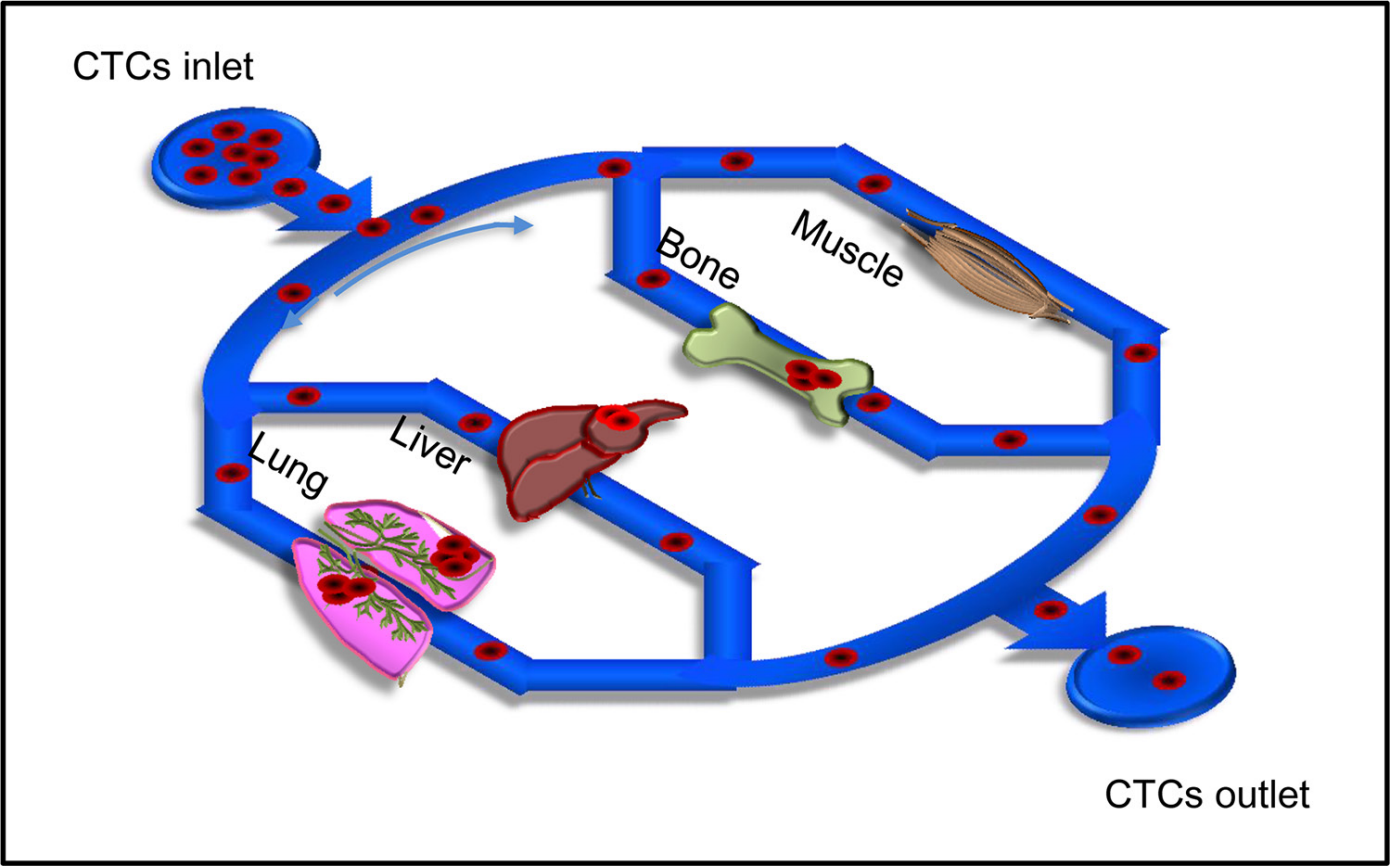
llustration of organ-specific metastasis of CTCs in the microfluidic chip CTCs were pumped over multiple artificial organs, including lung, liver, bone, and muscle cells in the biomimetic model, followed by quantitation of organ-specific metastasis of CTCs.

Biomimetic models that can reproduce complex and integrated physiological and pathological events are important alternatives to animal models in basic biological research and drug development. In the present study, we developed a microfluidic model that reproduced the organ-specific metastasis of CTCs (Figure 8). To reconstitute multiple organs on the microfluidic model, primary cells of mouse organs were isolated and cultured in organ chambers. These primary cells retained many characteristics of the original organs, such as secreting different levels of chemokines using the microfluidic model. Chemokines approximately 10,000 Da in size could diffuse from organ chambers into microchannels, to act as attractants of CTCs to facilitate the metastatic process.

Adhesion between flowing CTCs and the microvascular endothelium of specific organs is a key step in hematogenous metastasis [39, 40]. Shear stress generated by the flow can inhibit the adhesion between CTCs and endothelium to further decrease metastasis [41, 42]. To reproduce the *in vivo* flowing condition of CTCs, a physiological flow rate was used in the microfluidic model. Overall, multiple organs, microvessels with endothelium, and flowing CTCs were integrated in the microfluidic model, and the metastatic potentials of MCF7, MDA-MB-231, and ACC-M cells toward multiple organs could be assessed. The three tumor cell lines tested showed significantly higher metastatic potentials to lung, liver, and bone marrow cells than to muscle cells. This result was consistent with clinical observations. Together, the results demonstrated that the bimimetic multiple organ model provided a novel platform for the study of organ-specific metastasis of CTCs.

We wanted to determine if our microfluidic model had the physiological similarity to traditional metastatic animal models such as the nude mouse. Lung is the most common metastatic destination in many cancers. To establish the lung mestatasis animal model, tumor cells are injected into the mouse tail vein. Usally, it takes over 3 weeks to form detectable metastases as assessed by histological examination. We compared the metastatic capabilities of MCF7, MDA-MB-231, and ACC-M cells assessed by the microfluidic model with this traditional lung metastasis animal model. It was found that the results obtained by the microfluidic model were consistent with results obtained from the animal model. This result strongly suggested, at least for lung metastasis, that the microfluidic model had the potential to mimick the animal model. Furthermore, compared to the animal model, the microfluidic model was simpler to operate, less expensive, and significantly more time-saving, taking only 2 days to perform metastatic tests.

To investigate whether this microfluidic model could be used to rapidly and inexpensively screen drugs for their potential to inhibit metastasis, the ability of AMD3100 to inhibit MDA-MB-231 metastasis was directly compared using the microfluidic and animal models. AMD3100, also named Plerixafor, is an antagonist of CXCR4 which is clinically used to treat non-Hodgkin's lymphoma and multiple myeloma [43]. The results with both the microfluidic and animal models showed that AMD3100 was able to reduce the lung metastasis of MDA-MB-231. Because the adhesion of CTCs to endothelium were reproduced in the microfluidic model, agents targeting CTC adhesion can now be rapidly screened using this model. And the microfluidic model can be used in preclinical tests to assess the abilities of new compounds to inhibit several processes in organ-specific metastasis.

Regarding limitations of the present study, we only used the microfluidic model and animal models to compare the capability of cancer cells to metastasize to the lung. In animal models, metastasis is largely influenced by both the site of injection and the metastatic capacity of tumor cells. Injection routes include the lateral tail vein, plus other intraportal, intracarotid, and intracardiac sites. Tail vein injection is the most commonly used assay, and primarily leads to lung metastasis. Intraportal vein injection usually results in liver metastasis, while intracarotid injection leads to brain metastasis. Only intracardiac injection can result in metastasis to multiple organs (such as bone, liver, brain, and lung) because tumor cells are introduced into the arterial circulation. However, intracardiac injection is a difficult procedure and has a very high mortality rate, because injected tumor cells can induce a hypercoagulable state that leads to platelet consumption and thromboemboli formation [44]. Nonetheless, future studies should be conducted to compare the microfluidic model with other animal models of metastasis.

## MATERIALS AND METHODS

### Ethical statement

Animal experiments, transportation, and care were conducted in compliance with the relevant laws and the guidelines issued by the Ethical Committee of Dalian Medical University. The experimental groups had five mice per group and the control group had five mice. Animals were fed in the SPF level laboratory. They were sacrificed after 30 days.

### Additional methods

Additional methods involving fabrication of the microfluidic device, culture and labeling of cells, immunofluorescent staining, isolation of primary mouse cells, assessment of cell viability, and determination of CXCL12 secretion from primary cells can be found in the Supplementary Methods.

### Chemokine-induced metastasis assay using the microfluidic model

HUVECs have been previously reported to be served as the vascular endothelial barrier [29, 31, 45, 46]. Similarly, they were seeded onto the porous membrane of the device, which was pre-coated with BME (R&D Systems, Minneapolis, MN, USA), and cultured for 24 hours to mimic vascular endothelial barrier. CXCL12 as the member of chemokine family play an important role of metastasis. Different concentrations (0 ng/mL, 25 ng/mL, 50 ng/mL, and 100 ng/mL) of recombinant CXCL12 (R&D Systems) were loaded into each organ chamber and incubated for 5 hours. Tumor cells were cultured with serum-free medium for 5 hours, then harvested and loaded into the cell reservoir at a concentration of 2 × 10^6^ cells/mL. Then a syringe pump (Baoding Longer Precision Pump Co., Ltd, Baoding, China) was connected. The flow rate (750 nL/minute) was adjusted to almost the same as that of microvessels *in vivo*. Driven by the syringe pump, tumor cells flowed into the microchannels at a constant rate for 20 minutes, then the cells were incubated for another 10 minutes after stopping the pump. Phosphate-buffered saline (PBS) was used to remove loosely attached cells. At least three images were recorded randomly for each microchannel at 100× magnification using an inverted fluorescent microscope (Olympus IX71, Olympus Corp., Tokyo, Japan). Image-Pro^®^ Plus version 6.0 (Media Cybernetics, Inc. Silver Spring, MD, USA) software was used to count the number of tumor cells arrested on HUVEC cells in the microchannels. Each experiment was repeated at least eight times.

### Organ-specific metastasis assay using the microfluidic device

The organ chambers of the device were coated with collagen I (Life Technologies Corp., Gaithersburg, MD, USA) before cell seeding. Mixed primary cells isolated from specific organs of SD rats were then seeded into individual organ chambers. The total numbers of mixed primary cells are 4 × 10^5^ cells in individual organ chambers. HUVEC cells were seeded on the porous membrane, then the device was incubated at 37°C in 5% CO_2_ for 24 hours. Tumor cells were loaded into the cell reservoir at a concentration of 2 × 10^6^ cells/mL, and a syringe pump was connected with the syringe pump connector to drive the flow of tumor cells. The same assay steps were used to measure chemokine-induced metastasis.

### *In vivo* metastasis assay using a nude mouse model

BALB/c nude mice aged 4−5 weeks old (18 g, female, Dalian Medical University Laboratory Animal Center) were used to assess the *in vivo* metastatic capability of tumor cells. Mice were divided into three groups according to type of transplanted tumor cells: the MCF7 group, the MDA-MB-231 group, and the ACC-M group, with five mice in each group. Cells were harvested and resuspended at a concentration of 2 × 10^6^ cells/mL in PBS, then injected into the tail vein. The nude mice were raised in sterile conditions for 30 days, then euthanized and the lung tissues removed. Tissues were fixed in 10% formalin for 24 hours and embedded in paraffin. Sections of 4 μm thickness were prepared and stained with hematoxylin and eosin (H.E.). Immunohistochamical staining with anti-humna Pan CK (Merckmillipore, USA) was performed to detect the metastatic colonies. The images were recorded by a microscope (Olympus BX43, Olympus Corp., Tokyo, Japan). The images were recorded by a microscope (Olympus BX43, Olympus Corp., Tokyo, Japan). Metastatic rate and metastatic colony areas per lung section were used to evaluate the metastatic capability of each tumor cell line. Metastasis was regarded as positive even if only one metastatic colony was found in the lung. Metastatic rate was represented as the ratio of animals with positive metastasis in all experimental animals. High power view images (400×) were used to calculate metastatic colony areas. Firstly, the colonies were confirmed by H.E. and anti-human Pan cytokeratin immunostaining and circumscribed using Image-Pro^®^ Plus version 6.0 software. The metastatic areas were determined as the total areas of all metastatic colonies in the lung.

### Inhibition of lung metastasis by AMD3100 using the microfluidic device and mouse models

To inhibit lung metastasis *in vitro*, the anti-metastatic reagent AMD3100 (Sigma, St. Louis, MO, USA) was used. Different concentrations of AMD3100 (0 μg/mL, 0.5 μg/mL, 1 μg/mL, and 2 μg/mL) were introduced into the microchannels and incubated overnight. Tumor cells were resuspended at a concentration of 2 × 10^6^ cells/mL in culture medium with AMD3100 and loaded into the cell reservoir, followed by the same assay steps as previously described.

To inhibit lung metastasis *in vivo*, AMD3100 (Sigma) was used in a mouse model. Three hours after injection of tumor cells into the tail vein, five BALB/c-nude mice (4−5 weeks old, female) were subcutaneously treated with 1.25 mg/kg AMD3100, followed by AMD3100 treatment twice daily. After 30 days, the mice were euthanized, and lung tissues were dissected and submitted for histological examination.

### Statistical analysis

Statistical analyses were performed using SPSS version 13.0 for Windows (SPSS Inc., Chicago, IL, USA). The independent sample nonparametric-test was used to analyze comparisons of all data and were expressed as mean ± SE. Each experiment was conducted at least three times, and significance was identified as a *p* value of < 0.05.

## CONCLUSIONS

In summary, we have developed a microfluidic multiple organ model to reproduce organ specific metastasis of CTCs. Comparisons between this microfluidic model and the nude mouse model demonstrated that the microfluidic model has the similarity to the metastatic animal model. Overall, this multiple organ model shows a certain potential to provide an inexpensive and time-saving alternative to animal models, to predict the metastatic capabilities of different CTCs for specific organs, and to rapidly screen possible anti-metastatic drugs.

## SUPPLEMENTARY MATERIALS


