# Supplementary material for: A novel microfluidic model can mimic organ-specific metastasis of circulating tumor cells
Source: Oncotarget. 2016 May 15;7(48):78421–32. doi: 10.18632/oncotarget.9382 (PMC5346650; doi:10.18632/oncotarget.9382)
Supplement: Supplementary file 1 [file oncotarget-07-78421-s001.pdf]

A novel microfluidic model can mimic organ-specific metastasis of circulating tumor cells

Supplementary Materials

MATERIALS AND METHODS

Fabrication of the microfluidic device

The device is composed of a glass substrate and two layers of the polydimethylsiloxane (PDMS) membrane. The PDMS layer was fabricated by replicate molding the master, which was prepared by spin coating SU8-3035 negative photoresist (Microchem Corp.) onto a glass wafer, and then patterned by photolithography. Sylgard 184 PDMS base and curing agent (Sylgard Silicone elastomer 184, Dow Corning Corp.) were mixed thoroughly (10:1 by mass), degassed under vacuum, and poured onto the master. The polymer curing process was conducted in an oven for 1 hr at 80°C. After cooling, the PDMS layer was gently peeled off of the master and trimmed to size. Inlet and outlet holes were created by punching through the PDMS with a razor-sharp punch. We used two size puncher (1.5 mm and 3 mm diameter). To bond the second layer of PDMS and porous membrane (3 µm-pore size, 6~8µm-thick, Waterman) to the first layer PDMS, the PDMS prepolymer (5–10 µm thick) served as "glue" which was coated onto the surface of the first layer PDMS. The combined three parts were cured into an 80 ºC oven for 30 mins until the liquid prepolymer were completely polymerization. After heating-cured bonding, the two-layer PDMS membrane was bonded to a glass substrate irreversibly after oxygen plasma treatment for 90 s. Prior to use, the device was sterilized with UV light for 30 min.

Assess the function of HUVEC monolayer

To assess the tight junction of HUVECs, we did immunofluorescent assay of ZO-1. After fixed with 4% paraformaldehyde and blocked with 10% goat serum, the HUVECs were incubated with the rabbit anti-ZO-1 polyclonal antibody (1:50, Proteintech) at 4°C overnight and stained with secondary antibody next day.

In this study, we used CXCL12 as a chemoattractant through the HUVEC monolayer to induce the tumor cells. In order to assess the permeability of HUVEC monolayer, HUVEC cells were seeded in the microchannels pre-coated by BME. The 10 kDa (similar molecular weight to CXCL12) Fluorescein isothiocyanate–dextran (Sigma) were mixed with cell culture media at a concentration of 1 mM and loaded into bottom chambers to establish diffusion test across the endothelial monolayer. The normal cell culture media crossed the top channel with continuous perfusion (750 nl/min). From the 0 minute, the media of top channel were collected into centrifuge tubes in the 10 minutes, 30 minutes, 120 minutes and 240 minutes. After this operation, we monitored the intensity of fluorescence with Multi-Mode Microplate Reader (Synergy H1, Bio Tek.) in time.

Cell culture and labeling

Human Umbilical Vein Endothelial cell line (HUVEC) was provided from pharmaceutical college of Dalian medical university. Three types of tumor cell line were used in this study. A salivary gland adenoid cystic carcinoma cell line (ACC-M) was presented by Dr Wang (Guangzhou, China). Human breast cancer cell lines (MCF-7 and MBA-MD-231) were purchased from ATCC. HUVEC, ACC-M, and MCF7 were cultured at 37°C with 5% CO2 and 95% relative humidity in DMEM/F12 (Hyclone) supplemented with 10% FBS (Hyclone), 100 U/mL penicillin, and 100 U/mL streptomycin. MDA-MB-23 was cultured in L-15 medium (Hyclone) supplemented with 7% FBS and 100 U/mL penicillin, 100 U/mL streptomycin. To visualize tumor cell arrestment on HUVEC, ACC-M cells were labeled by CellTrackerTM Red CMPTX (Invitrogen) and breast cancer cells were labeled by CellTracker™ Green CMFDA (Invitrogen).

Isolation of murine primary cells

Primary murine pulmonary cells were isolated from fresh lung of male Sprague-Dawley (SD) rats (160–180 g). The tissue was cut into pieces (1 mm^3) on the ice and digested by using combined digestion of trypsin and type I collagenase (Invitrogen) in 37ºC. Then the primary pulmonary cells were cultured in DMEM/F12 containing 10% FBS, 100 U/mL penicillin, and 100 U/mL streptomycin.

Primary murine hepatocytes were isolated from male SD rats (160–180 g) using a two-step perfusion procedure. First, after sterilizing, the rat was anaesthetized and an incision was made in the midline of abdomen. Second, a 24G indwelling needle was inserted into the portal vein and the liver was infused with D-Hank's (5.4 mM KCL, 0.4 mM KH2PO4, 137 mM NaCL, 4.2 mM NaHCO3, mM $Na_2HPO_4 \cdot 12H_2O$, 5.6 mM D-glucose, pH = 7.4)

and 0.02% EDTA to remove blood cells. The velocity of perfusion was 20 mL/min over 15 minutes. Third, enzyme perfusion was performed with collagenase IV solution in 37°C with 20 mL/min over 20 minutes. Following collagenase perfusion, rat liver cells are dissociated and filtrated through 100 μm cell strainer (BD, USA). The cell suspension was centrifuged three times at 50 × g for 3 min. At last the cells was suspended and cultured with high-glucose DMEM supplemented with, 5% FBS, 100 U/mL penicillin, and 100 U/mL streptomycin, 10 mM nicotinamide.

Primary murine bone marrow-derived cells (BMC) were isolated from the long bones of SD rats. The femurs, tibiae, and humeri were dissected using sterile forceps and surgical scissor. The bone tissues were placed in culture dish with cold PBS solution. The bone epiphyses were cut off and the marrow cavities were flushed using injector with DMEM/F12. The washing solution was harvested and centrifuged at 3000 rpm/min for 10 min. Cell pellets were resuspended in 1 × PBS. In order to avoid the contamination of the palate and red blood cells, the Percoll (Pharmacia) was used for discontinuous gradients centrifugation. The centrifugal force was 1500 rpm/min for 20 min. The cells stayed at the surface of 60% Percoll. Then the BMC were collected and cultured in DMEM/F12 with 10% FBS, 100 U/mL penicillin, and 100 U/mL streptomycin.

Primary murine muscle cells were isolated from skeletal muscles. The hind limbs' muscles were stripped from male SD rat (200 g) and immersed in cold PBS. Then the muscles were minced into pieces (1 mm^3) and cultured in a dish stationarily with DMEM-F12 containing 10% FBS, 100 U/mL penicillin, and 100 U/mL streptomycin. Generally, primary skeletal muscle cells grew out from the muscle segments after 3 days. These cells were trypsinized when they reached confluence.

Cell viability assay

The viability of primary cells was assessed. Hoechst 33342 (Molecular Probes, 4 μg/mL) and Rhodamine-123 (Sigma, 2 μg/mL) were dissolved in serum-free culture medium and incubated cells for 40 min at 37ºC. Then the cells were rinsed with PBS and incubated with Propidium Iodide (PI, Sigma, 2 μg/mL) for 5min at 37ºC. After PBS washing, the cells were imaged under an inverted fluorescent microscope (Olympus, IX71).

Determination of CXCL12 secretion from primary cells

CXCL12 secretion by primary cells was determined by Rat stromal cell derived factor 1α ELISA Kit (Cusabio). Supernate was collected and centrifuged to remove particulates. The processes of incubation were all at 37ºC for 1 h. TMB chromogenic reaction was performed at 37ºC for 15 min and protected from light. The result was read at 450 nm in a plate reader (Multiskan MK3, Thermo).

Immunofluorescent staining

Cells were culture on coverslips. Before immunofluorescent staining, the coverslips were washed with PBS three times and cells were fixed with 4% paraformaldehyde for 20 min at 4ºC, rinsed with PBS. Then cells were blocked with 10% goat serum for 30 minutes. Cells were incubated with rabbit anti-CXCR4 polyclonal antibody (10 μg/ml; Merck Millipore) at 4ºC overnight. After washed with PBS, cells were incubated with FITC-conjugated mouse anti-rabbit antibody (Invitrogen) for 30 minutes and washed with PBS. Before the fluorescence imaging, the cell nucleus stained with DAPI (1:2000; Life Technology) and wash with PBS buffer. Images were record with a Two-photon confocal microscope (Leica CP5-II).

Flow cytometry

Cell surface expression of CXCR4 receptor on MCF-7, MDA-MB-231 and ACC-M cell lines were analyzed by flow cytometry analysis. Cells were harvested from dishes non-enzymatically and resuspended at 1×10^6 cells/mL in ice-cold buffer (PBS with 0.1% BSA). Each type of cell lines was divided into three groups, including blank control group, negative control group and test group. The test group and negative control group prepared 1×10^6 cells in 1mL buffer were incubated with 10 μl PE-conjugated mouse antihuman CXCR4 antibody 12G5 (BD Biosciences) or PE-conjugated mouse IgG2A (BD Biosciences) at room temperature for 30 min in the dark. However, the blank control group did not do any dispose. After incubation, cells were washed and adjusted to a concentration of 1×10^7 cells in 1 mL PBS and analyzed on a FACS Aria II (Becton Dickinson) immediately.
